# Integrin Beta 5 Is a Prognostic Biomarker and Potential Therapeutic Target in Glioblastoma

**DOI:** 10.3389/fonc.2019.00904

**Published:** 2019-09-20

**Authors:** Lu-yang Zhang, Qing Guo, Ge-fei Guan, Wen Cheng, Peng Cheng, An-hua Wu

**Affiliations:** ^1^Department of Neurosurgery, The First Hospital of China Medical University, Shenyang, China; ^2^College of Applied Technology, China Medical University, Shenyang, China

**Keywords:** integrin, ITGB5, Glioblastoma, prognosis, brain cancer

## Abstract

**Background:** Glioblastoma (GBM) is the most lethal cancer of the central nervous system. Integrin beta 5 (*ITGB5*) is thought to be involved in intercellular signal transduction and regulation of tumor initiation and progression. However, the function of *ITGB5* in GBM is not known.

**Methods:** To address this question, we evaluated the expression level of *ITGB5* in clinical specimens by immunohistochemistry and western blotting, as well as the association between *ITGB5* expression and GBM patient survival using data from Chinese Glioma Genome Atlas and The Cancer Genome Atlas. The biological function of *ITGB5* in GBM was investigated by Gene Ontology, gene set enrichment, and *in vitro* loss-of-function experiments using glioma cells.

**Results:** Among integrin family members, *ITGB5* showed the greatest difference in expression between low-grade glioma and GBM. Elevated ITGB5 expression was highly correlated with glioma progression and a mesenchymal subtype and poor survival in GBM patients. *ITGB5* was found to be associated with regulation of the immune response and angiogenesis in GBM, and was required for migration and invasion of glioma cells and tube formation by endothelial cells.

**Conclusions:** These data indicate that ITGB5 can serve as a predictive biomarker for GBM patient survival and is a potential therapeutic target in GBM treatment.

## Introduction

Glioblastoma (GBM) is the most common primary malignant brain tumor in adults. Even when different treatment approaches such as surgical resection, chemotherapy, and radiotherapy are combined, tumor recurrence is inevitable and the prognosis of GBM patients is extremely poor, with a median survival of 12–15 months (1–3). Significant efforts are being made to identify GBM surface molecules and pathways that can be targeted by therapeutics (4).

Integrins are a group of integral transmembrane heterodimers with many functions including cell adhesion in the extracellular matrix and acting as receptors for various physiological ligands (5). Aberrant integrin expression profiles are often observed in aggressive tumors such as breast cancer; therefore, these proteins are potential drug targets (5). Integrins are the major determinant of the invasive phenotype of glioma (4, 6). Two integrin α subunits, ITGA6 and ITGA7, are key receptors in glioma stem-like cells (4, 7). Thus, antagonizing integrins may be a useful strategy for preventing GBM progression, and characterizing the expression patterns of integrins and related signaling pathways in glioma—especially GBM—can provide insight into the molecular mechanisms of tumor recurrence and treatment resistance in malignant glioma.

Integrin β5 (ITGB5) encodes a subunit of integrin that can interact with several integrin α chains. ITGB5 mediates transforming growth factor (TGF)β-induced epithelial-mesenchymal transition (EMT) and contributes to the tumorigenic potential of breast cancer cells (8, 9). A recent study reported that ITGB5 promotes tumorigenesis in hepatocellular carcinoma by interacting with β-catenin (10). In addition, ITGB5 was enriched in liver metastatic pancreatic cancer exosomes (11). This indicated its potential role in intercellular communication during tumor progression and metastasis. In GBM, ITGB5 was shown to regulate GBM-infiltrating macrophages in the local microenvironment via modulation of osteopontin (12). However, the role of ITGB5 in the tumor microenvironment is not fully understood.

Characterizing ITGB5 expression and how this is related to patient prognosis may be useful for the development of more effective treatment strategies for GBM and for predicting the outcome of glioma patients. To this end, in the present study we examined the expression of ITGB5 in clinical glioma samples and its relationship with the outcome of glioma patients. We found that ITGB5 overexpression in GBM was associated with poor survival. Furthermore, ITGB5 not only promoted the migration and invasion of glioma cells but also regulated the function of endothelial cells. Conversely, ITGB5 silencing decreased the expression of EMT markers in glioma cells.

## Materials and Methods

### Clinical Specimens and Ethics Approval

Clinical specimens were collected at the First Hospital of China Medical University from January 2011 to February 2018 (non-tumor, *n* = 5; grade II, *n* = 7; grade III, *n* = 17; and grade IV, *n* = 61). There were 53 grade IV (GBM) samples for which patient survival information was available that were used for immunohistochemical analysis. A total of 17 clinical specimens for western blotting were collected at the First Hospital of China Medical University from June 2017 to February 2018 (non-tumor, *n* = 3; grade II, *n* = 4; grade III, *n* = 4; and grade IV, *n* = 6). Histological diagnoses were confirmed by two neuropathologists according to the 2016 World Health Organization classification. The study protocol was approved by the Ethics Committee of the First Hospital of China Medical University.

### Datasets for ITGB5 Expression and Survival Analyses

Gene expression profiles from Chinese Glioma Genome Atlas (CGGA) and The Cancer Genome Atlas (TCGA) were used for *ITGB5* expression and patient survival analyses. The CGGA RNA Sequencing (RNAseq) dataset and associated clinical information (http://www.cgga.org.cn) included 310 samples (grade II, *n* = 105; grade III, *n* = 67; and GBM, *n* = 138). The *ITGB5* expression and clinical data in TCGA and Ivy GAP were extracted from GlioVis (http://gliovis.bioinfo.cnio.es/) (13). TCGA RNAseq dataset included 625 samples (grade II, *n* = 227; grade III, *n* = 243; and GBM, *n* = 155); TCGA-4502A mRNA microarray dataset comprised 488 GBM samples. The Ivy GAP RNAseq dataset was used to analyze *ITGB5* expression in different GBM regions.

### Cell Lines and Culture

LN229 cells were provided by Professor Tao Jiang (Department of Molecular Neuropathology, Beijing Neurosurgical Institute). Human umbilical vein endothelial cells (HUVECs) were provided by Professor Xin Meng (Department of Biochemistry, China Medical University). LN229 cells and HUVECs were maintained in Dulbecco's modified Eagle's medium (DMEM; Gibco, Grand Island, NY, USA) supplemented with 10% fetal bovine serum (FBS; Gibco) and 1% penicillin/streptomycin (Gibco) at 37°C with 5% CO_2_. Patient-derived primary glioma cells (PGC1228) were cultured from fresh glioma samples according to a protocol approved by the Ethics Committee of the First Hospital of China Medical University. The cells were cultured in Roswell Park Memorial Institute (RPMI)-1640 medium (Gibco) containing 10% FBS and 1% penicillin/streptomycin (Gibco) at 37°C and 5% CO_2_, and the established cell line was authenticated.

### Immunohistochemistry and Western Blotting

Immunohistochemistry and immunoblotting were performed as described in our previous study (14). Antibody information is shown in Table S1.

#### RNA Isolation and Reverse Transcription Quantitative PCR (RT-qPCR)

After RNA isolation and first-strand cDNA synthesis, RT-qPCR was performed with SYBR Green Master Mix (Takara Bio, Otsu, Japan) according to the manufacturer's instructions. The forward and reverse primer sequences were as follows: *ITGB5*, GGAAGTTCGGAAACAGAGGGT and CTTTCGCCAGCCAATCTTCTC; and *glyceraldehyde 3-phosphate dehydrogenase*, GGAGCGAGATCCCTCCAAAAT and GGCTGTTGTCATACTTCTCATGG).

### Receiver Operator Characteristic (ROC) Curve

The ROC curve was plotted and the area under the ROC curve of each cutoff was determined as previously described (15, 16).

### Gene Ontology (GO) Analysis

Genes whose expression was correlated with that of *ITGB5* were defined by a Pearson *r* > 0.3 and *P* < 0.05 in two GBM datasets (CGGA RNAseq and TCGA RNAseq). GO analysis was performed using DAVID 6.8 [https://david.ncifcrf.gov/tools.jsp (17)].

### Gene Set Enrichment Analysis (GSEA) and Gene Set Variation Analysis (GSVA)

Patients were stratified into two groups according to the median *ITGB5* mRNA expression level. GSEA was performed to determine whether the identified sets of genes showed statistically significant differences between the two groups based on normalized enrichment score and false discovery rate (18). GSVA (http://www.bioconductor.org) was performed to further verify whether the genes were correlated with specific signaling pathways (19).

### Tumor Purity, Immune and Stromal Scores, and Microenvironment Cell Populations-Counter (MCP-Counter)

Tumor purity and immune and stromal scores were calculated as previously described (20). Eight immune and two non-immune stromal cell populations [immune cells: T cells, cluster of differentiation (CD)8+ T cells, natural killer (NK) cells, cytotoxic T lymphocytes, B cells, monocytes, myeloid dendritic cells, and neutrophils) and stromal cell populations (endothelial cells and fibroblasts) were evaluated by the MCP-counter method (21).

### Small Interfering (si)RNA and Cell Transfection

Specific siRNAs targeting *ITGB5* (siITGB5) and a negative control siRNA (siNC) were synthesized by Sangon Biotech (Shanghai, China). The *ITGB5* siRNA sense and antisense sequences were as follows: si*ITGB5*-368, GCUCGCAGGUCUCAACAUATT and UAUGUUGAGACCUGCGAGCTT; and si*ITGB5*-1218, GCCAACGAGUACACUGCAUTT and AUGCAGUGUACUCGUUGGCTT. The siRNAs were transfected using Lipofectamine 3000 reagent (Life Technologies, Carlsbad, CA, USA) according to the manufacturer's instructions.

#### *In vitro* Cell Proliferation Assays

Cell growth was evaluated with the MTS assay (Promega, Madison, WI, USA) according to the manufacturer's instructions.

### Cell Migration and Invasion Assays

Transwell inserts with a pore size of 8 μm (Corning Inc., Corning, NY, USA; 3422) were used for *in vitro* cell migration and invasion assays. To assess cell migration, LN229 and PGC1228 cells transfected with siITGB5 or siNC were resuspended in DMEM containing 0.2% FBS and seeded into the upper chambers of the transwell insert at a density of 2 × 10^4^/200 μl. A 600-μl volume of DMEM containing 20% FBS was added to the lower chamber and the number of cells that migrated into the lower chamber was counted. For the invasion assay, LN229 and PGC1228 cells transfected with siITGB5 or siNC were resuspended as described above and seeded into the upper chamber of the insert that was pre-coated with 500 ng/ml Matrigel solution (BD Biosciences, Franklin Lakes, NJ, USA) at a density of 4 × 10^4^/200 μl. A 600-μl volume of DMEM containing 20% FBS was added to the lower chamber. After 24 h, cells on the upper side of the membrane were removed with a cotton swab; the membrane was fixed with methanol and stained with 1% crystal violet solution, and the number of cells on the lower side of the membrane in five random high-power fields per well was counted under a microscope.

### Conditioned Medium

Conditioned medium was obtained by culturing equal numbers of treated glioma cells in DMEM or RPMI-1640 medium at 37°C and 5% CO_2_ for 24 h. The media were passed thought a 0.2-μm pore filter prior to use in experiments (22).

### Tube Formation Assay

The Matrigel assay was used to evaluate *in vitro* angiogenesis based on the quantification of tube formation (23). Briefly, each well of a 96-well culture plate was coated with 100 μl of Matrigel and the plate was incubated for 30 min at 37°C. HUVECs were resuspended in tumor-conditioned medium at a density of 2.0 × 10^5^ cells/ml. A 100-μl volume of the cell suspension was seeded in each Matrigel-coated well of the 96-well culture plate, followed by incubation at 37°C for 12 h. Images were acquired at 100 × magnification using a fluorescence microscope (DP71; Olympus, Tokyo, Japan), and the total number of branched tubules was quantified (22).

### Statistical Analysis

Data were analyzed with SPSS v.20 (SPSS Inc., Chicago, IL, USA) and Prism 7 software (GraphPad Inc., La Jolla, CA, USA) software. Heat maps were generated with R v.3.4.2 (https://www.r-project.org/). Statistical significance was defined as *P* < 0.05. Differences between and among groups were evaluated with the two-tailed *t*-test and by one-way analysis of variance followed by Turkey *post-hoc* test, respectively. Univariate and multivariate Cox regression analyses were performed with R v.3.4.2. A Kaplan-Meier survival analysis was used to estimate the survival distribution, followed by the log-rank test to evaluate differences among stratified groups using the median value as a cutoff. *ITGB5* copy number variation (CNV) frequency in glioma was evaluated with GISTIC2.0 (24); a locus with a GISTIC value ≥ 1 or ≤ -1 was defined as an amplification or deletion, respectively.

## Results

### ITGB5 Is Differentially Expressed Between Low-Grade Glioma (LGG) and GBM

**Figure 1:**
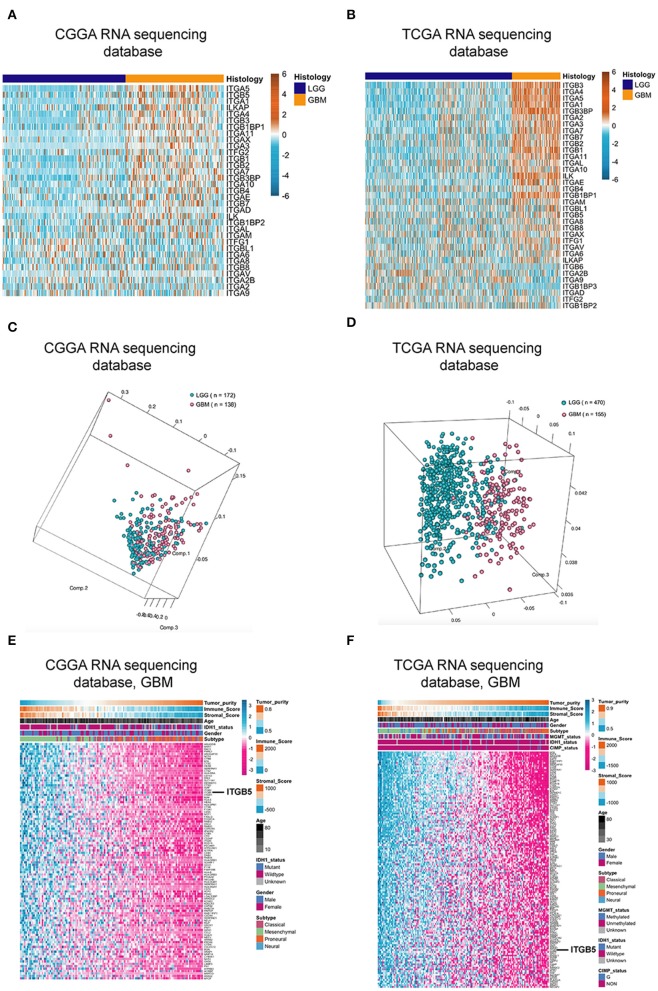
*ITGB5* expression in glioma. **(A,B)** Heatmaps of expression patterns of integrin family genes in glioma based on CGGA **(A)** and TCGA RNAseq **(B)** data. **(C,D)** PCA based on vesicle-related genes in CGGA **(C)** and TCGA **(D)** stratifies LGG and GBM. **(E,F)** Heatmaps showing the positive correlation between vesicle-related genes and tumor purity and immune and stromal scores (**E**, CGGA; **F**, TCGA RNAseq). *r* > 0.45 or <-0.45 (Pearson's correlation analysis).

Over 20 integrin proteins have been identified, including 18 α and eight β integrin subunits (5). The importance of integrins in cancer biology makes these proteins attractive therapeutic targets (25, 26). In this study, we analyzed the expression of genes encoding integrin family members in glioma using CGGA and TCGA RNAseq datasets and found that *ITGB5* showed the greatest difference in expression between LGG and GBM (Figures 1A,B). We investigated the *ITGB5* copy number variation (CNV) frequency in glioma and observed the following: grade II: amplification, 0.89% and deletion, 5.80%; grade III: amplification, 2.88% and deletion, 7.82%; and GBM: amplification, 18.00% and deletion, 6.67%) (TCGA RNAseq dataset); and GBM: amplification, 12.61% and deletion, 10.04% (TCGA 4502A microarray dataset) (Table S2). These data indicate that no mutations are present in *ITGB5* in GBM.

### ITGB5 Overexpression Is Associated With Tumor Purity and Immune and Stromal Scores

Extracellular vesicle (EV)-mediated cell-to-cell communication plays an important role in cancer (27, 28). We compiled a list of EV-related genes from intercellular and secretory vesicle gene sets (http://software.broadinstitute.org/gsea/) (Table S3), and a principal component analysis (PCA) revealed that these genes can distinguish GBM from LGG (Figures 1C,D). We examined the correlation between extracellular vesicle-related genes and tumor purity as well as immune and stromal scores in CGGA and TCGA datasets and determined that *ITGB5* is the only gene that is significantly correlated with all three factors along with unfavorable survival of GBM patients based on a univariate Cox regression analysis (*P* < 0.01; Figures 1E,F; Figure S1; Table S4).

### Elevated ITGB5 Expression Is Associated With Progressive Malignancy and a Mesenchymal Subtype in Glioma

**Figure 2:**
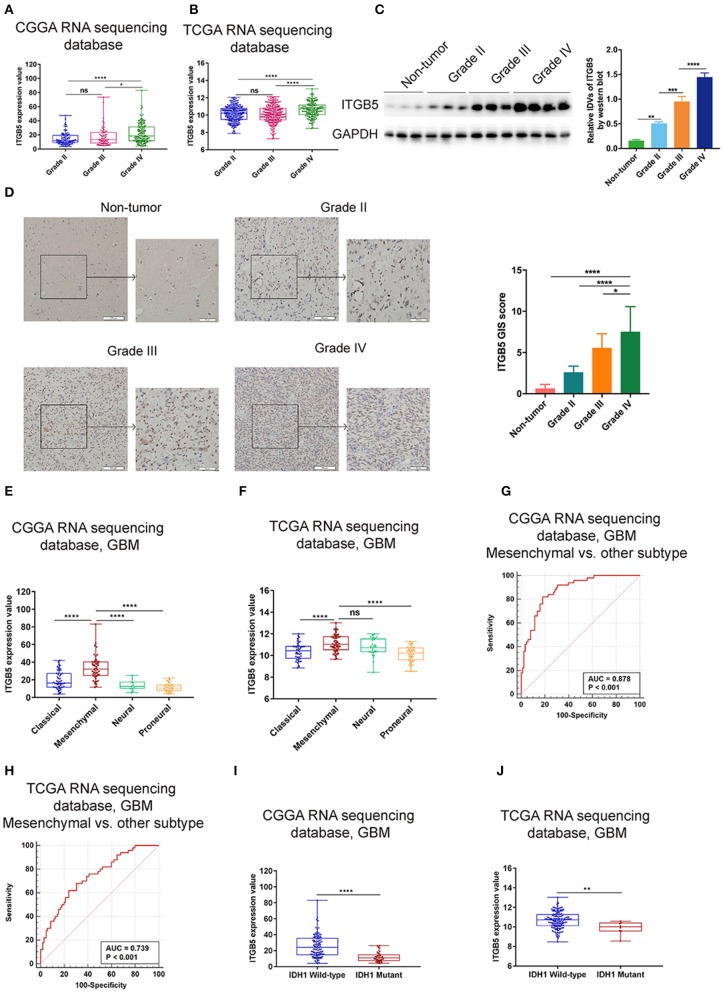
Elevated *ITGB5* expression is associated with progressive malignancy and a mesenchymal subtype in glioma. **(A,B)** *ITGB5* expression according to glioma grade (**A**, CGGA RNA-seq; **B**, TCGA RNA-seq). ns, *P* > 0.05; *P < 0.05; **** *P* < 0.0001 (one-way analysis of variance [ANOVA]). **(C)** *ITGB5* expression is elevated according to glioma grade, as determined by western blotting. **P < 0.01; ***P < 0.001; ****P < 0.0001 (one-way ANOVA). **(D)** Representative images (left panel) of ITGB5 expression in clinical specimens and quantification of staining intensity (right panel). *P < 0.05; ****P < 0.0001 (one-way ANOVA). **(E,F)** ITGB5 expression is highest in the mesenchymal subtype of GBM (**E**, CGGA RNA-seq; **F**, TCGA RNA-seq). ns, *P* > 0.05; ****P < 0.0001 (one-way ANOVA). **(G,H)** ROC curve for evaluating the sensitivity and specificity of ITGB5 as a diagnostic marker for the mesenchymal subtype of GBM vs. other subtypes (**G**, CGGA RNAseq, area under the ROC curve [AUC]: 0.878, *P* < 0.001; **H**, TCGA RNAseq, AUC: 0.739, *P* < 0.001). **(I,J)** ITGB5 expression is elevated in *IDH1*-wild-type as compared to *IDH1*-mutant GBM (**I**, CGGA RNAseq; **J**, TCGA RNAseq, **P < 0.01; ****P < 0.0001 [*t*-test]).

To further investigate the expression profile of *ITGB5* in glioma, we analyzed *ITGB5* expression in different glioma grades. ITGB5 levels increased with tumor grade (Figures 2A,B), and western blot and immunohistochemical analyses of clinical specimens showed that ITGB5 was overexpressed in GBM as compared to non-tumor and grades II and III glioma tissue (Figures 2C,D). We then analyzed ITGB5 expression in different GBM subtypes using CGGA and TCGA datasets and found that *ITGB5* was more closely associated with the mesenchymal phenotype (Figures 2E,F; Figure S2A), suggesting that it could serve as a diagnostic marker for this subtype (Figures 2G,H; Figure S2B). Moreover, according to data from the Ivy database, *ITGB5* was overexpressed in areas of microvascular proliferation relative to other regions of the tumor (Figure S2C) and in isocitrate dehydrogenase (*IDH*)*1*-wild-type as compared to *IDH1*-mutant GBM (Figures 2I,J; Figure S2D). However, there was no significant difference in *ITGB5* expression between GBM patients with and those without *O6-methylguanine DNA methyltransferase* promoter methylation (Figures S2E,F). Thus, elevated *ITGB5* expression is associated with progressive malignancy in glioma and is specific to the mesenchymal subtype.

### High ITGB5 Levels Predict Poor Prognosis in GBM

**Figure 3:**
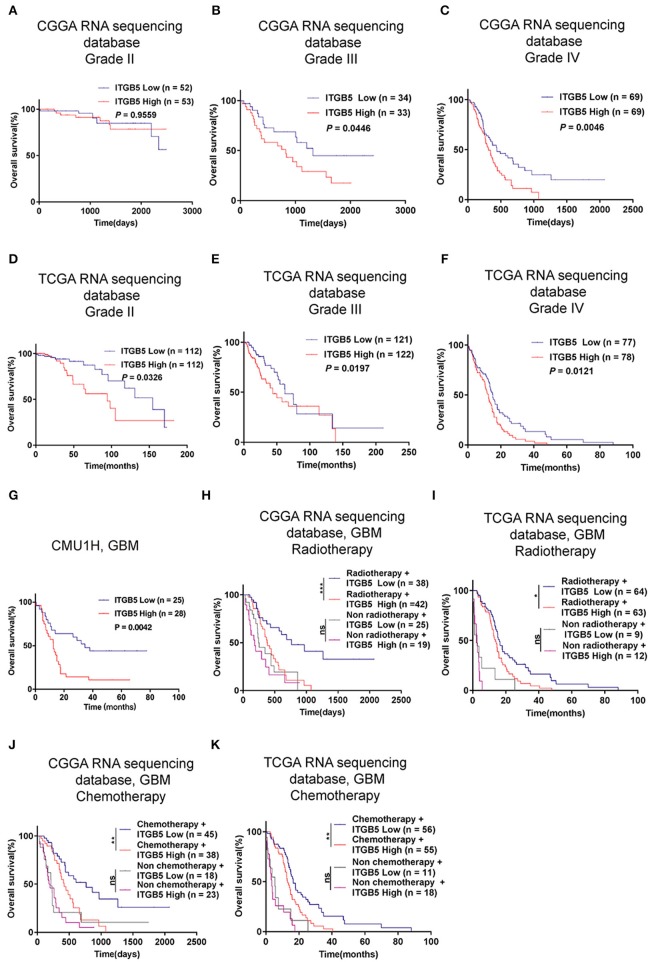
*ITGB5* overexpression is a prognostic biomarker in GBM. **(A–F)**, Kaplan-Meier analysis of the association between *ITGB5* expression and patient survival according to glioma grade (**A**, CGGA grade II; **B**, CGGA grade III; **C**, CGGA grade IV; **D**, TCGA grade II; **E**, TCGA grade III; **F**, TCGA grade IV). **(G)** Kaplan-Meier analysis of the correlation between high ITGB5 expression and patient prognosis in GBM (ITGB5 high vs. low, *P* = 0.0042; log-rank test). **(H–K)** Results of the Kaplan-Meier analysis demonstrating that high ITGB5 expression is associated with poor survival in GBM patients receiving radiotherapy (**H**, CGGA RNAseq; **I**, TCGA RNAseq) or chemotherapy (**J**, CGGA RNAseq; **K**, TCGA RNAseq). ns, *P* > 0.05; *P < 0.05; **P < 0.01; ***P < 0.001 (log-rank test).

Given the association between high ITGB5 expression and glioma grade, we speculated that ITGB5 could be a prognostic biomarker for glioma outcome. To test this hypothesis, we examined the correlation between ITGB5 expression and the survival of glioma patients. Elevated ITGB5 expression was associated with a favorable outcome for glioma patients (Figures 3A–F; Figure S3A). We also analyzed the relationship between ITGB5 expression and the survival of 53 GBM patients and determined that patients with higher ITGB5 levels had poorer outcomes compared to those with a lower ITGB5 expression level (Figure 3G). Furthermore, among GBM cases in CGGA and TCGA who underwent radio- and chemotherapy, those with higher ITGB5 expression had a significantly shorter survival time than those with lower expression (Figures 3H–K; Figures S3B,C). To assess the correlation between ITGB5 expression and the clinical characteristics in GBM, we performed univariate and multivariate Cox regression analyses with the clinical characteristics of age, *IDH1* mutation status, radiotherapy, and chemotherapy as variables. The results showed that ITGB5 is an independent prognostic factor for overall survival in GBM (Table 1). Taken together, these results suggest that ITGB5 is a useful biomarker for predicting survival in GBM patients.

**Table 1:** Univariate and multivariate Cox regression analyses of *ITGB5* in CGGA and TCGA RNAseq datasets and overall survival of GBM patients.

**Variable**	**Univariate regression**		**Multivariate regression**	
	**HR**	***P*-value**	**HR**	***P*-value**
**CGGA RNAseq, GBM**				
Age	1.0050	0.5665	0.96348	0.0029
*IDH1*	0.7071	0.2017	0.61566	0.1449
Radiotherapy	0.4119	0.0002	0.48658	0.0053
Chemotherapy	0.3359	0.0000	0.31579	0.0000
*ITGB5*	1.0189	0.0095	1.02759	0.0039
**TCGA RNAseq, GBM**				
Age	1.0267	0.0010	1.01027	0.2589
*IDH1*	0.2110	0.0081	0.30458	0.0023
Radiotherapy	0.2351	0.0000	0.86749	0.6752
Chemotherapy	0.4507	0.0001	0.35453	0.1654
*ITGB5*	1.3776	0.0027	1.32495	0.0132

### ITGB5 May Be Involved in Immune Regulation and Angiogenesis in the GBM Microenvironment

**Figure 4:**
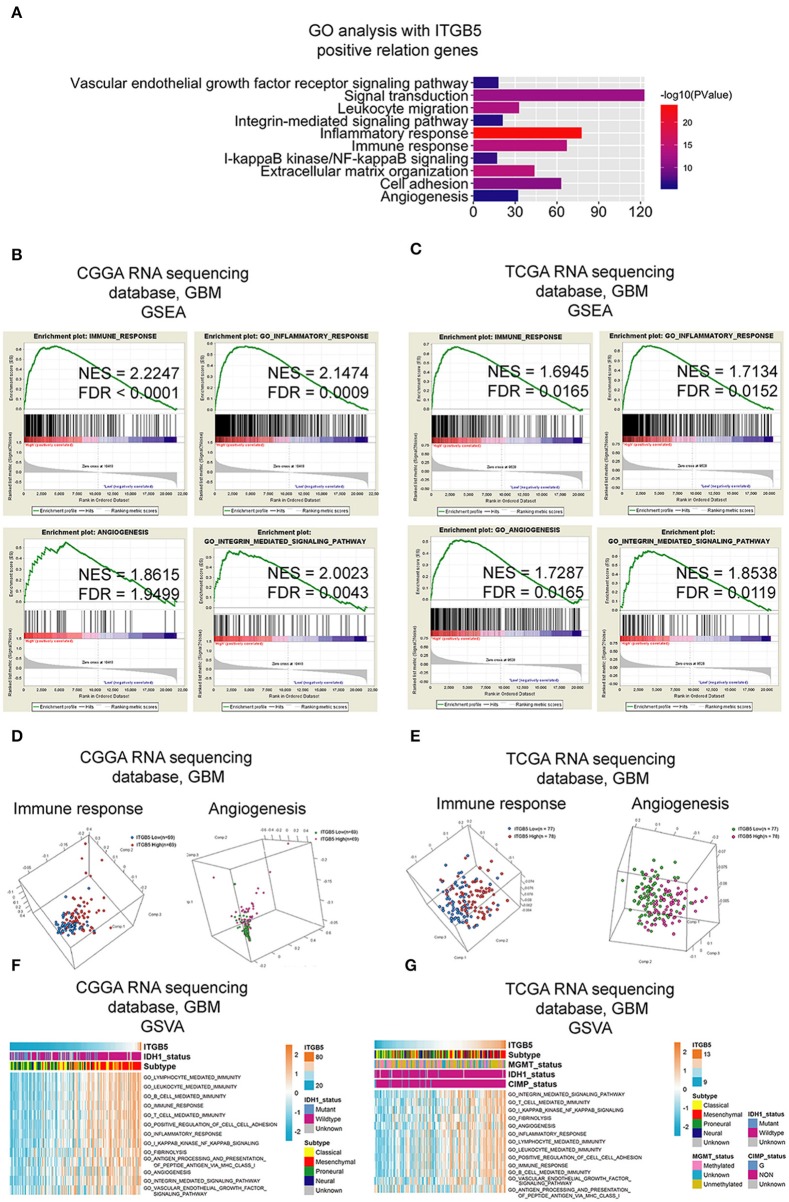
Functional analysis of signaling pathways associated with high *ITGB5* expression level in GBM. **(A)** GO analysis of genes positively correlated with *ITGB5* based on CGGA and TCGA RNAseq datasets (Pearson's correlation analysis). **(B,C)** GSEA analyses of CGGA **(B)** and TCGA **(C)** GBM RNAseq datasets showing the enrichment of immune response, inflammatory response, angiogenesis, and integrin-mediated signaling pathways in patient samples with high *ITGB5* expression. **(D,E)** PCA analysis based on CGGA **(D)** and TCGA **(E)** GBM RNAseq datasets shows that high ITGB5 expression can stratify immune response and angiogenesis signaling. **(F,G)** GSVA showing the correlation between high *ITGB5* levels and immune response and angiogenesis-related signaling pathways.

To clarify the function of ITGB5 in GBM, we compiled a list of 1,043 genes whose expression is correlated with a high *ITGB5* expression level based on CGGA and TCGA GBM RNAseq datasets (*r* > 0.3, *P* < 0.05; Table S5). A GO analysis revealed an association between *ITGB5* expression and the GO terms vascular endothelial growth factor signaling pathway, migration, inflammatory response, immune response, cell adhesion, and angiogenesis (Figure 4A). As mentioned above, *ITGB5* was the only gene correlated with tumor purity and immune and stromal scores in CGGA and TCGA datasets (*r* > 0.45 or < -0.45; Table S4). Based on this observation, we carried out GSEA to investigate the function of ITGB5 in GBM. The immune response, inflammatory response, angiogenesis, and integrin-mediated signaling were associated with elevated ITGB5 expression (Figures 4B,C). Additionally, PCA based on CGGA and TCGA GBM RNAseq datasets demonstrated that high *ITGB5* levels can stratify immune response and angiogenesis signaling (Figures 4D,E). Consistent with these results, GSVA revealed that ITGB5 contributes to the regulation of local immune response, cell adhesion, and vascular endothelial growth factor signaling (Figures 4F,G). Thus, ITGB5 is involved in the immune response and angiogenesis in the GBM microenvironment.

### ITGB5 Is Required for Glioma Cell Migration and Invasion and for Tube Formation by Endothelial Cells

**Figure 5:**
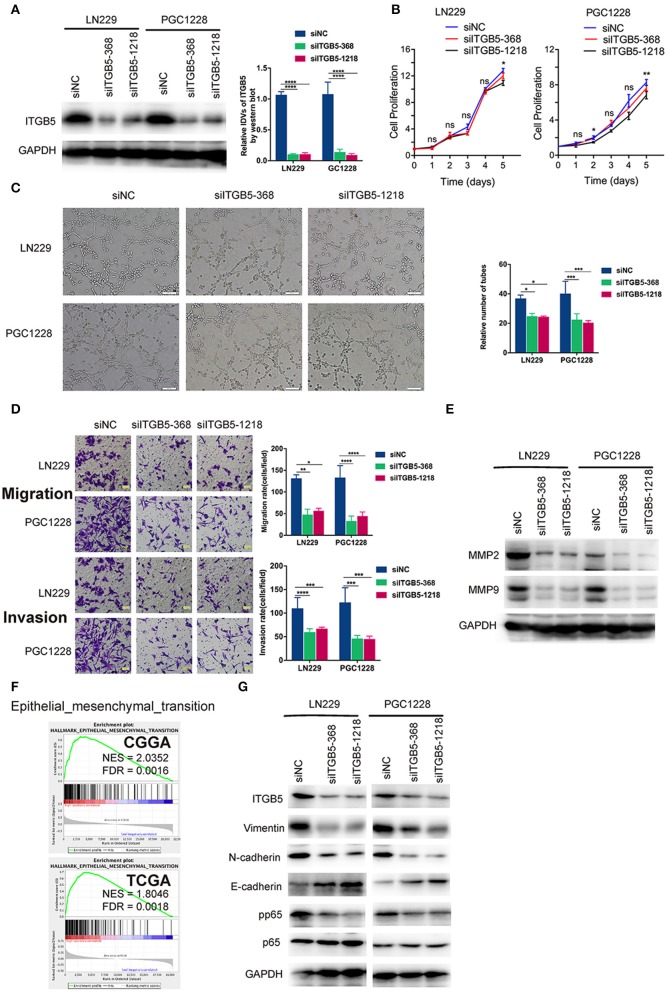
*ITGB5* silencing attenuates the migration and angiogenesis of glioma cells and inhibits epithelial-mesenchymal transition marker expression. **(A)** Representative western blots (left panel) and analysis (right panel) of glioma cells transfected with indicated siRNAs targeting *ITGB5* (si*ITGB5*-368 and si*ITGB5*-1218) or siNC. **(B)** The effect of *ITGB5* silencing on the growth of LN229 and PGC1228 cells. **(C)** *ITGB5* knockdown suppresses tube formation by endothelial cells treated with conditioned medium from cultured glioma cells. **(D)** Glioma cell migration (upper panel) and invasion (lower panel) are inhibited by *ITGB5* knockdown. **(E)** Western blot analysis of glioma cells transfected with indicated siRNAs showing that *ITGB5* silencing attenuates MMP2 and MMP9 expression. **(F)** GSEA of CGGA and TCGA RNAseq datasets reveals the enrichment of epithelial–mesenchymal transition-related genes in specimens with high ITGB5 levels. **(G)** Western blot analysis of glioma cells transfected with indicated siRNAs targeting *ITGB5* (si*ITGB5*-368 and si*ITGB5*-1218) or siNC. *ITGB5* silencing suppressed EMT marker expression. ns, *P* > 0.05; *P < 0.05; **P < 0.01; ***P < 0.001; ****P < 0.0001 (one-way analysis of variance [ANOVA]).

To examine the functions of ITGB5 in GBM in greater detail, we used two *ITGB5*-specific siRNAs to knock down *ITGB5* expression in a stable glioma cell line (LN229) and a primary glioma cell line derived from a clinical GBM specimen (PGC1228) (Figure 5A; Figure S4). ITGB5 silencing did not significantly affect the growth of LN229 at day 1, 2, 3, and 4 (Figure 5B, left panel). For PGC1228, there was significantly difference at day 2 and day 5, and there was no significant difference at day1, 3, and 4 (Figure 5B, right panel); however, the tube formation capacity of endothelial cells induced with glioma cell-conditioned medium as well as glioma cell migration and invasion were inhibited (Figures 5C,D). The expression of matrix metalloproteinase (MMP)2 and MMP9—two proteins that regulate the migration and invasion of glioma cells—was also attenuated by ITGB5 knockdown (Figure 5E). In addition, GSEA revealed that EMT signaling was increased in association with high ITGB5 expression (Figure 5F). Consistent with this observation, western blot analysis of glioma cells transfected with siRNAs targeting *ITGB5* showed that the expression of several EMT markers including vimentin, N-cadherin, and phosphorylated p65 was downregulated by ITGB5 depletion (Figure 5G). These data indicate ITGB5 not only regulates the migration and invasion of glioma cells but also tube formation capacity in endothelial cells.

### ITGB5 Expression Is Associated With Non-tumor Immune and Stromal Cell Populations in the Glioma Microenvironment

**Figure 6:**
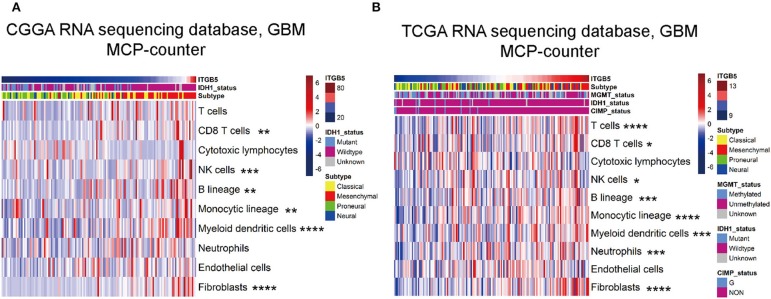
Correlation between *ITGB5* expression and non-tumor immune and stromal cell populations in the GBM microenvironment. Cell populations were quantified with MCP-counter (**A**, CGGA GBM RNAseq; **B**, TCGA GBM RNAseq). *P < 0.05; **P < 0.01; ***P < 0.001; ****P < 0.0001 (Pearson's correlation analysis).

We evaluated the association between *ITGB5* and the immune and stromal cell populations in the GBM microenvironment with the MCP-counter method. The results showed that CD8+ T cell, NK cell, fibroblast, B cell, monocyte, and myeloid dendritic cell numbers were positively associated with ITGB5 expression (Figures 6A,B), providing further evidence for the regulatory role of ITGB5 in the local tumor microenvironment.

## Discussion

Despite the use of multimodal treatment strategies, the survival time of most GBM patients is <2 years (1). Reliable prognostic biomarkers for this disease are needed in order to improve patient outcome and monitor the response to standard radio-and chemotherapy. Moreover, the characterization of molecular changes has important clinical implications for predicting glioma outcome (29). Our analyses showed that ITGB5 was overexpressed in GBM, especially in the mesenchymal subtype. Another novel finding of this study was that high ITGB5 levels were independently correlated with shorter survival time in patients. These data highlight the value of ITGB5 as a predictive biomarker in GBM.

A typical feature of GBM is disruption of the local microenvironment (20). Our bioinformatic analyses revealed that high ITGB5 expression is correlated with integrin-related, immune, and angiogenesis signaling, suggesting that ITGB5 may play an important regulatory role in the GBM microenvironment. Tumor-associated macrophages have been implicated in brain tumor angiogenesis and resistance to anti-angiogenic therapies, in part due to their ability to modulate vessel integrity and function (30–32). We show that *ITGB5* expression is positively correlated with the number of monocyte lineage cells, which may reflect the mechanism by which ITGB5 regulates the tumor microenvironment. This result is consistent with a recent report, which indicated that the heterodimer of ITGB5 and ITGAV expressed by GBM-infiltrating macrophages constituted a major OPN receptor and mediated the recruitment of macrophages (12). In comparison with non-tumor tissue, glioma, especially GBM, have an elevated expression level of ITGB5 (Figure 2C). This may indicate the potential of ITGB5 as a therapeutic target in the treatment of GBM. Considering ITGB5 expressed in normal tissue and non-tumor cells, further study is needed to investigate whether systematic blockade of ITGB5 may lead to potential side effects, like immunosuppression.

To investigate the function of ITGB5 in GBM, we performed knockdown experiments using siRNAs and found that glioma cell growth was unaffected by *ITGB5* depletion, although their migration and invasion were reduced. Moreover, the decreased capacity for endothelial tube formation caused by ITGB5 knockdown indicates that ITGB5 is required for angiogenesis, which may be promoted by the release of factors via extracellular vesicles. However, further studies are needed to investigate this possibility.

In summary, our study shows that a high expression level of *ITGB5* is an indicator of progressive malignancy in glioma and predicts unfavorable outcome in GBM patients, even after radiotherapy. ITGB5 not only influences the migration and invasion of glioma cells, but is also involved in regulating the immune response and angiogenesis in the tumor microenvironment. Thus, ITGB5 is a useful prognostic biomarker and potential therapeutic target in GBM.

## Data Availability

The datasets generated for this study are available on request to the corresponding author.

## Ethics Statement

The studies involving human participants were reviewed and approved by the Ethics Committee of the First Hospital of China Medical University. The patients/participants provided their written informed consent to participate in this study.

## Author Contributions

QG, PC, and AW conceived and designed the study. LZ, QG, and WC carried out the experiments and collected data and performed the bioinformatics analysis. PC, QG, GG, and AW drafted the manuscript. PC and AW obtained funding for the study. All authors read and approved the final manuscript.

### Conflict of Interest Statement

The authors declare that the research was conducted in the absence of any commercial or financial relationships that could be construed as a potential conflict of interest.

## Supplementary Material

Figure S1 Heatmaps of correlations between intercellular vesicle-related gene expression and tumor purity and immune and stromal scores based on TCGA 4502A mRNA microarray data. *r* > 0.45 or < -0.45 (Pearson's correlation analysis).

Figure S2 *ITGB5* expression in glioma. **(A)** *ITGB5* expression in different grades of glioma based on TCGA 4502A mRNA microarray data. ^****^*P* < 0.0001 (one-way ANOVA). **(C)** *ITGB5* expression in the Ivy GAP RNAseq dataset. ^**^*P* < 0.01; ^****^*P* < 0.0001 (one-way ANOVA). **(B)** ROC curve for evaluating the sensitivity and specificity of *ITGB5* as a diagnostic marker for the mesenchymal subtype of GBM as compared to the other subtypes (TCGA 4502A mRNA microarray, area under the ROC curve [AUC]: 0.757; *P* < 0.001). **(D)** ITGB5 expression is elevated in *IDH1*-wild-type as compared to *IDH1*-mutant GBM (TCGA 4502A mRNA microarray; *t*-test). **(E,F)** ITGB5 expression in GBM patients with or without *O6-methylguanine DNA methyltransferase* (*MGMT*) promoter methylation. (**E**, TCGA RNAseq; **F**, TCGA 4502A microarray). ns, *P* > 0.05 (*t*-test).

Figure S3 *ITGB5* is a prognostic biomarker in GBM. **(A)** *ITGB5* expression is associated with unfavorable prognosis in GBM (TCGA 4502A mRNA microarray). *P* = 0.0008 (log-rank test). **(B,C)** High *ITGB5* expression predicts the response of GBM patients to radiotherapy **(B)** and chemotherapy **(C)** (TCGA 4502A mRNA microarray) (^*^*P* < 0.05, ^**^*P* < 0.001, log-rank test).

Figure S4 *ITGB5* silencing has no effect on glioma cell growth. **(A)** Representative RT-qPCR analysis of glioma cells transfected with indicated siRNAs targeting *ITGB5* (siITGB5-368 and siITGB5-1218) or control siRNA (siNC). ^****^*P* < 0.0001 (one-way ANOVA).

Table S1 Antibodies used in this study.

Table S2 *ITGB5* copy number variation frequency in TCGA.

Table S3 Intercellular vesicle-related genes.

Table S4 Correlation analysis of intercellular vesicle-related genes with tumor purity and immune and stromal scores in CGGA and TCGA datasets (Pearson's correlation analysis).

Table S5 Genes positively correlated with high *ITGB5* expression in CGGA and TCGA GBM RNAseq datasets.
