# Supplementary figures and images for: Integrin Beta 5 Is a Prognostic Biomarker and Potential Therapeutic Target in Glioblastoma
Source: Front Oncol. 2019 Sep 20;9:904. doi: 10.3389/fonc.2019.00904 (PMC6764112; doi:10.3389/fonc.2019.00904)

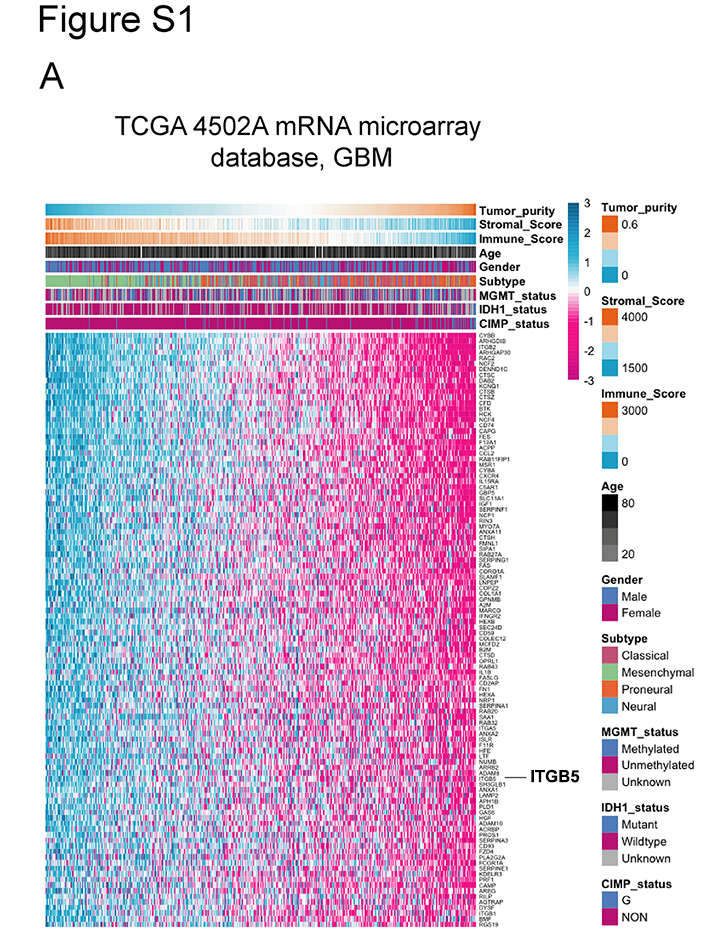

Supplement: Figure S1 — Heatmaps of correlations between intercellular vesicle-related gene expression and tumor purity and immune and stromal scores based on TCGA 4502A mRNA microarray data. r > 0.45 or < -0.45 (Pearson's correlation analysis). [file Image_1.TIF]

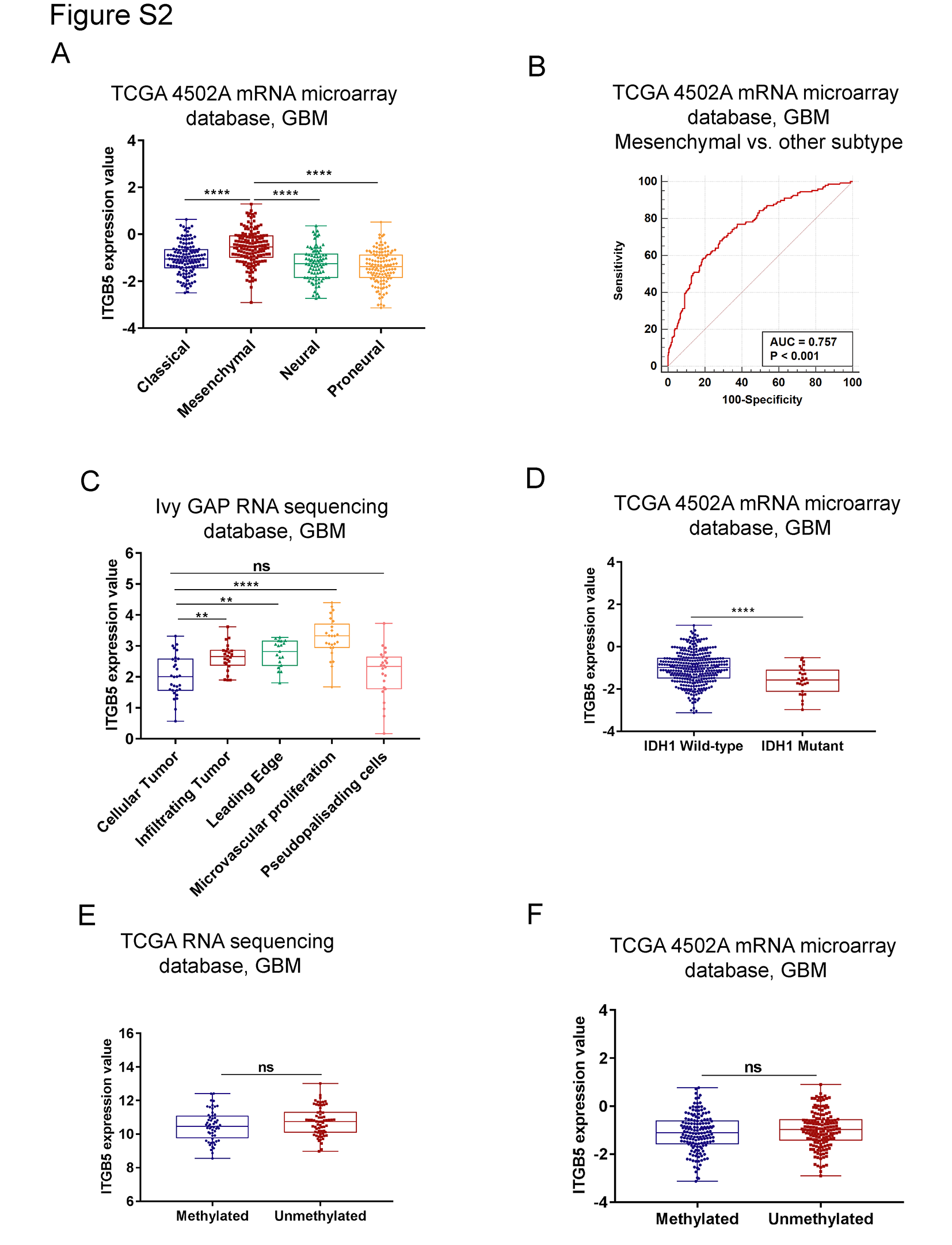

Supplement: Figure S2 — ITGB5 expression in glioma. (A) ITGB5 expression in different grades of glioma based on TCGA 4502A mRNA microarray data. ****P < 0.0001 (one-way ANOVA). (C) ITGB5 expression in the Ivy GAP RNAseq dataset. **P < 0.01; ****P < 0.0001 (one-way ANOVA). (B) ROC curve for evaluating the sensitivity and specificity of ITGB5 as a diagnostic marker for the mesenchymal subtype of GBM as compared to the other subtypes (TCGA 4502A mRNA microarray, area under the ROC curve [AUC]: 0.757; P < 0.001). (D) ITGB5 expression is elevated in IDH1-wild-type as compared to IDH1-mutant GBM (TCGA 4502A mRNA microarray; t-test). (E,F) ITGB5 expression in GBM patients with or without O6-methylguanine DNA methyltransferase (MGMT) promoter methylation. (E, TCGA RNAseq; F, TCGA 4502A microarray). ns, P > 0.05 (t-test). [file Image_2.TIF]

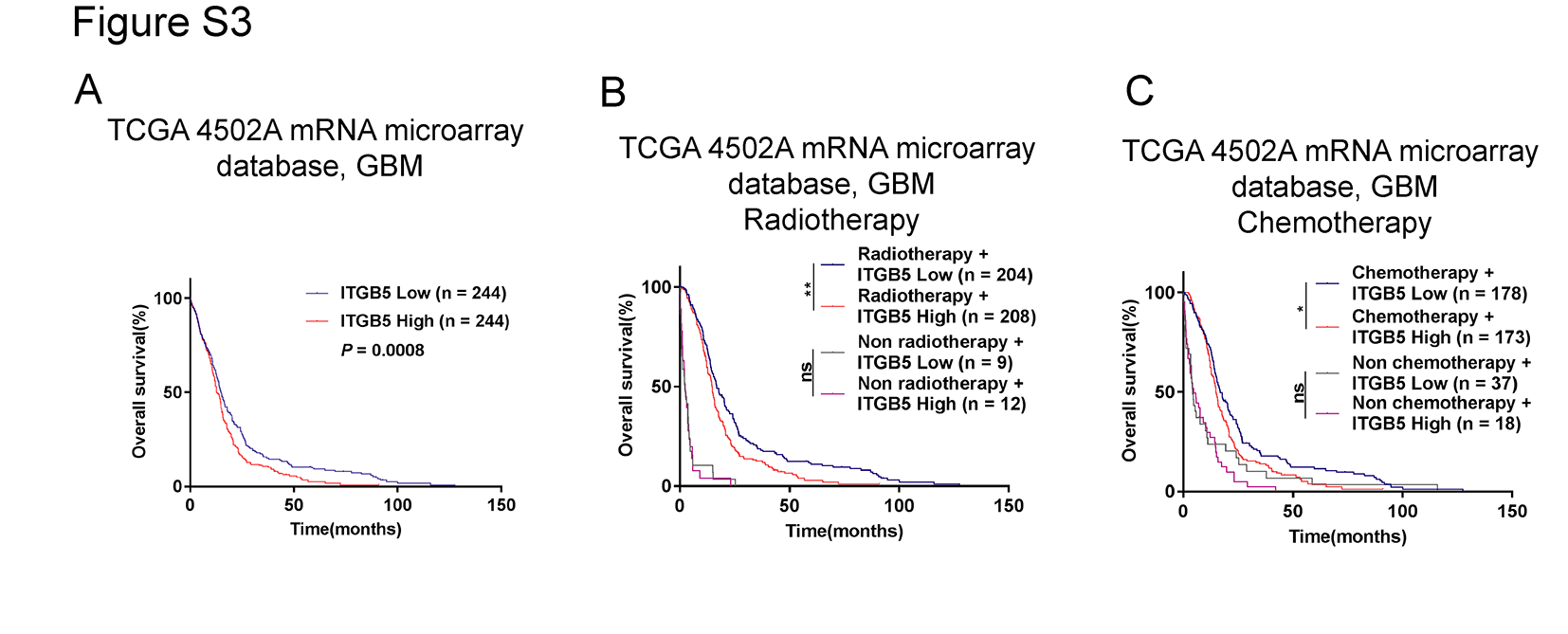

Supplement: Figure S3 — ITGB5 is a prognostic biomarker in GBM. (A) ITGB5 expression is associated with unfavorable prognosis in GBM (TCGA 4502A mRNA microarray). P = 0.0008 (log-rank test). (B,C) High ITGB5 expression predicts the response of GBM patients to radiotherapy (B) and chemotherapy (C) (TCGA 4502A mRNA microarray) (*P < 0.05, **P < 0.001, log-rank test). [file Image_3.TIF]

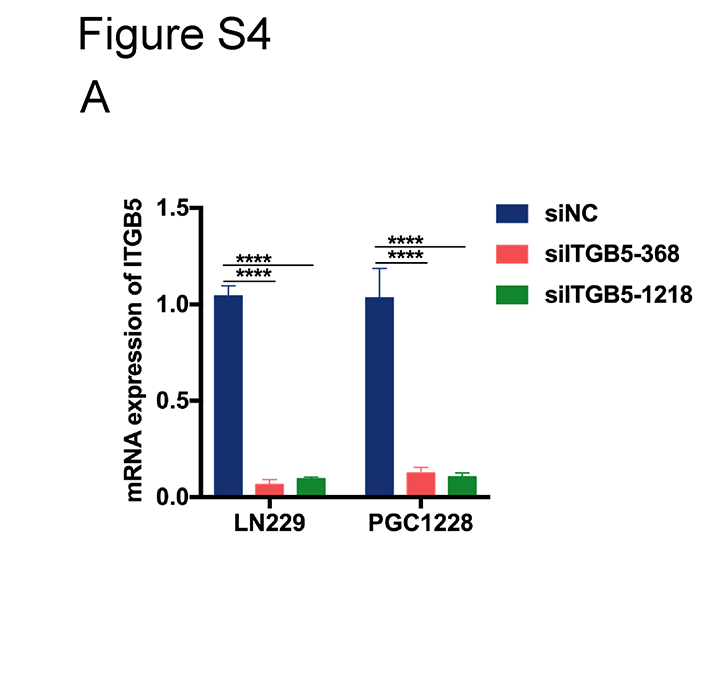

Supplement: Figure S4 — ITGB5 silencing has no effect on glioma cell growth. (A) Representative RT-qPCR analysis of glioma cells transfected with indicated siRNAs targeting ITGB5 (siITGB5-368 and siITGB5-1218) or control siRNA (siNC). ****P < 0.0001 (one-way ANOVA). [file Image_4.TIF]
